# Photocatalytic and electrocatalytic approaches towards atmospheric nitrogen reduction to ammonia under ambient conditions

**DOI:** 10.1186/s40580-019-0182-5

**Published:** 2019-04-25

**Authors:** Jude John, Dong-Kyu Lee, Uk Sim

**Affiliations:** Department of Materials Science & Engineering, Chonnam National University, Gwangju, 61186 Republic of Korea

**Keywords:** Haber–Bosch process, Nitrogen reduction reaction (NRR), Photoelectrochemical, Electrochemical

## Abstract

Ammonia production is essential for sustaining the demand for providing food for the growing population. Being a great source of hydrogen, it has significant potential in turning out to be a viable candidate for the future hydrogen economy. Ammonia has a high hydrogen content of about 17.6 wt %, is easier to liquefy and is produced in large quantities. Even though large-scale production of ammonia is significant globally, it is used predominantly as a fertilizer. It used also as a transport fuel for vehicles because of its low carbon emissions. Ammonia as an energy storage media is realized in many countries with infrastructure for transportation and distribution already put into place. Currently, the Haber–Bosch process is employed globally in industrial ammonia production and is a high energy expending process requiring large capital investment. In realizing a much economic pathway given the large-scale ammonia production growth forecast, it is necessary to seek new and improved methods for large-scale ammonia production. Amongst them, photoelectrochemical and electrochemical approaches stand as most promising towards nitrogen reduction to ammonia owing to their design features, lesser complexity, and economical in terms of the conventional ammonia production system. Several catalyst materials are investigated which include metal oxides, metals sulfides, carbon-based catalysts, and metal nitrides are all currently being pursued better utilization of their catalytic property towards nitrogen fixation and the minimization of the competing hydrogen evolution reaction (HER). In this article, we have summarized the design and reaction mechanisms for photoelectrochemical and electrochemical nitrogen fixation with the inherent challenges and material- related issues in realizing the Nitrogen Reduction Reaction (NRR).

## Introduction

Ammonia (NH_3_) synthesis by the Haber–Bosch process is regarded as one of the most significant realizations of the 20th century, contributing to a global production of over 150 million tons of NH_3_ yearly, practically 80% of which is in fertilizer production [1]. Global demands for NH_3_ production are also likely to increase with its future additional uses such as a carbon-free fuel and particularly as hydrogen storage system owing to its high energy density (15.3 MJ/L), easy handling and storage [2]. The current system of NH_3_ production through the Haber–Bosch process is energy-intensive with global consumption of 1 to 2% of the total annual energy production [3]. The reason for these high level of energy requirements arises from exothermic N_2_ reduction reaction as in (1), requires elevated reaction temperatures (~ 500 °C) and high pressures (> 200 atm) and most importantly the need for large volumes of H_2_ gas. The potential issue that arises from the usage of natural gas as a source for H_2_ is the substantial levels of CO_2_ emissions due to its dependence on the natural gas produced through steam reforming [4, 5]. From a thermodynamic standpoint, the NRR is favored much by high pressures and low temperature but in terms of kinetics which requires higher operating temperatures for achieving realistic NH_3_ production rates [1].

1 $${\text{N}}_{ 2} + {\text{ 3 H}}_{ 2} \leftrightarrow {\text{ 2NH}}_{ 3} - 4 5. 9 {\text{ kJ mol}}^{ - 1}$$

The fundamental bottleneck in realizing electrochemical reduction of nitrogen can is understood better from the thermodynamic constraints imposed by the intermediate reactions. The NRR equilibrium potentials for different products generated during NRR is as follows:

2 $${\text{N}}_{ 2} + {\text{ 6H}}^{ + } + {\text{ 6e}}^{ - } \leftrightarrow {\text{ 2NH}}_{ 3} \left( {\text{g}} \right),{\text{ E}}^{0} = \, + 0. 5 5 {\text{ V vs NHE}}$$

3 $$2 {\text{H}}^{ + } + {\text{ 2e}}^{ - } \leftrightarrow {\text{ H}}_{ 2} ,{\text{ E}}^{0} = \, 0{\text{ V vs SHE at pH }} = \, 0$$

4 $$\begin{aligned} {\text{N}}_{ 2} + {\text{ 6H}}_{ 2} {\text{O }} + {\text{ 6e}}^{ - } \leftrightarrow {\text{ 2NH}}_{ 3} + {\text{ 6OH}}^{ - } \hfill \\ {\text{E}}^{0} = \, - 0. 7 3 6 {\text{ V vs SHE at pH }} = { 14} \hfill \\ \end{aligned}$$

5 $${\text{N}}_{ 2} + {\text{ 2H}}^{ + } + {\text{ 2e}}^{ - } \leftrightarrow {\text{ N}}_{ 2} {\text{H}}_{ 2} \left( {\text{g}} \right),{\text{ E}}^{0} = \, - 1. 10{\text{ V vs RHE}}$$

6 $${\text{N}}_{ 2} + {\text{ 4H}}^{ + } + {\text{ 4e}}^{ - } \leftrightarrow {\text{ N}}_{ 2} {\text{H}}_{ 4} \left( {\text{g}} \right),{\text{ E}}^{0} = \, - 0. 3 6 {\text{ V vs RHE}}$$

7 $${\text{N}}_{ 2} + {\text{ H}}^{ + } + {\text{ e}}^{ - } \leftrightarrow {\text{ N}}_{ 2} {\text{H}},{\text{ E}}^{0} = \, - 3. 2 {\text{ V vs RHE}}$$

8 $$\begin{aligned} {\text{N}}_{ 2} + {\text{ 4H}}_{ 2} {\text{O }} + {\text{ 6e}}^{ - } \leftrightarrow {\text{ N}}_{ 2} {\text{H}}_{ 4} + {\text{ 4OH}}^{ - } , \hfill \\ {\text{E}}^{0} = \, - 1. 1 6 {\text{ V vs SHE at pH }} = { 14} \hfill \\ \end{aligned}$$

9 $${\text{N}}_{ 2} + {\text{ e}}^{ - } = {\text{ N}}_{ 2}^{ - } \left( {\text{aq}} \right),{\text{ E}}^{0} = \, - 3. 3 7 {\text{ V vs RHE at pH }} = { 14}$$

The electrochemical reduction of N_2_ to NH_3_ is comparable to H_2_ evolution with the following equilibrium potentials. While this is in realizing the fact that H_2_ is the main side product during NRR in aqueous electrolytes. Regardless of these NRR takes place via multiple proton-electron transfer reactions, wherein several intermediates are involved. Equation (7) indicates the difficulty in the addition of the first H atom as a much negative redox potential is required. A significantly higher pH for Eq. (8) required for the reaction to compete with Eq. (7). Furthermore, the addition of second H atom is much more complex than the addition of the third H atom, thus requiring a larger reduction potential for the reduction of two-electron and four-electron processes when compared to reduction involving six-electron. (Eqs. (5), (6) and (8)). The thermodynamic difficulty for the N_2_ hydrogenation is further clear from the much negative reduction potentials for the intermediates. Since multiple catalytic reactions associated are with the formation of intermediates, this imposes a much critical limitation to the thermodynamics of NRR [4, 6].

NH_3_ besides being used as a fertilizer is currently pursued as a potential energy storage medium analogous to its high hydrogen content compared with that of methanol [7]. NH_3_ is expected to play a crucial role in the future hydrogen economy [8]. It is of great importance to developing an environmentally benign method for NH_3_ production. Electrocatalytic and photocatalytic approaches are indeed regarded as energy-efficient and offer an environmentally friendly approach towards NH_3_ production, as these processes are generally carried out under ambient conditions using solar and wind energy which are renewable energy resources [9] For example, solar cells and wind turbines can power electrolytic cells for N_2_ reduction, and similarly light (photo) driven N_2_ reduction can directly access sunlight as a potential light source wherein these two strategies can complement each other for minimizing the carbon emissions by establishing a much realistic NH_3_ production compared to the traditional approaches [10, 11]. The sole idea of electrocatalytic and photocatalytic NRR is entirely reliant on catalysts which form the core for the entire NRR [12–14].

Screening of catalysts based on the composition and structure through experimental and theoretical approaches have over time has been done, and many of these electro/photocatalysts have already been designed and fabricated for NH_3_ production under ambient conditions [15]. For realizing a suitable electrocatalyst for electrocatalytic NRR, over the past few years, some of the highly researched materials include noble metals, non-noble metals based, and conducting polymer-based electrocatalysts is reported over the past for electrocatalytic NRR operated under ambient conditions. Recently, the focus has shifted towards more potential electrocatalysts including transition metals with flat and stepped surfaces and metal nitrides, which have been explored much in much detail through theoretically calculating the free energy changes after finding the possible intermediates formed on these surfaces [16].

The discovery of TiO_2_ as a photocatalytic material for splitting water through irradiating ultraviolet light by Fujishima and Honda completely changed the perspective at which water-splitting viewed in the past [17]. Not until 1977 when Guth and Schrauzer reported that electron–hole pairs were generated by the absorption of light by rutile TiO_2_ to reduce N_2_ to NH_3_. Here they also required the use of ultraviolet light to excite the photocatalyst [18]. The solar irradiance observed on the earth’s surface comprises about 5% ultraviolet radiation (< 400 nm), visible light forms 50% (400–800 nm) and lastly infrared light constitutes for almost 45% (> 800 nm). However, the generated NH_3_ is oxidized immediately to nitrate making it difficult to generate NH_3_ photocatalytically. In the case of photocatalysts, much importance is given specifically to three classes which include inorganic hybrids [19], biomimetic chalcogels [20], and inorganic semiconductor [21] based electrodes. Among these hydrogen-terminated diamond [22], and black silicon [23] have been employed in the conversion of N_2_ to NH_3_ under ambient conditions. Electrocatalysts and photocatalysts play vital roles in N_2_ fixation to NH_3_, for which we would like to discuss the involved mechanisms. Understanding the underlying mechanism of the NRR is crucial for designing or fabricating efficient catalysts, and with the right knowledge, it would be appropriate to fine-tune or modify the properties of the existing electro/photocatalysts.

In this review, we would like to address the key reaction mechanisms involved in realizing electrocatalytic and photocatalytic NRR under ambient conditions from a futuristic perspective. We begin by introducing the underlying reactions mechanisms employed for electrocatalytic and photocatalytic N_2_ reduction to NH_3_ on a heterogeneous catalyst surface. When discussing the reaction mechanisms involved in realizing N_2_ reduction to NH_3_, it is necessary to identify the bottlenecks that hinder the natural process. Finally, we list the most promising state-of-the-art electro/photocatalysts reported so far in this area.

## Reaction mechanisms for electrochemical NRR

**Fig. 1:**
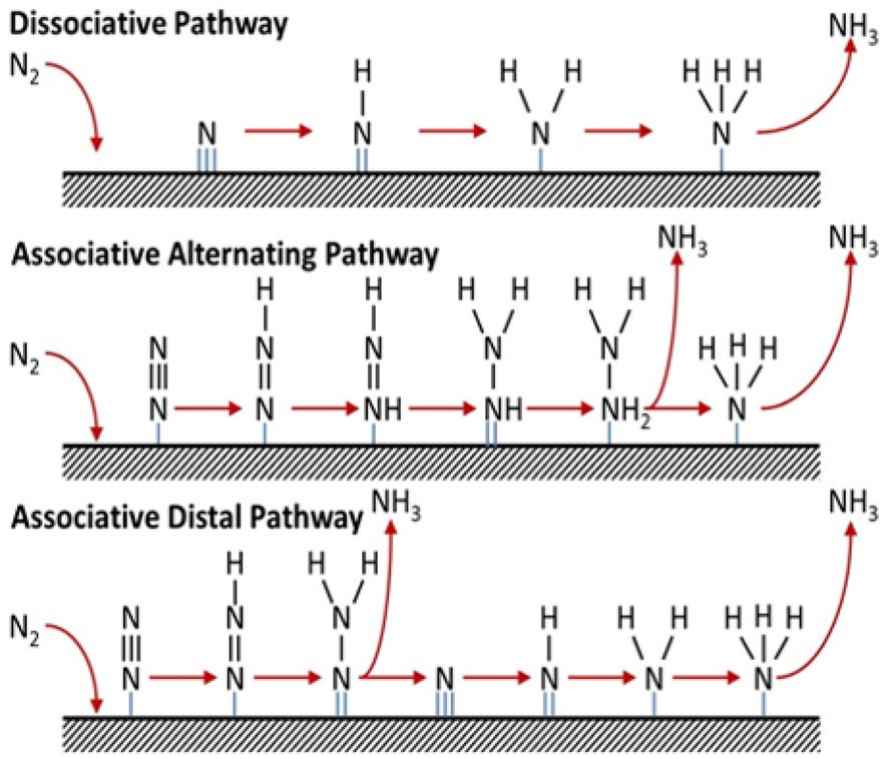
Reaction mechanism for catalytic conversion of N_2_ to NH_3_ on heterogeneous catalysts (Reproduced with permission [24] *Copyright 2016, Elsevier*)

**Scheme 1:**
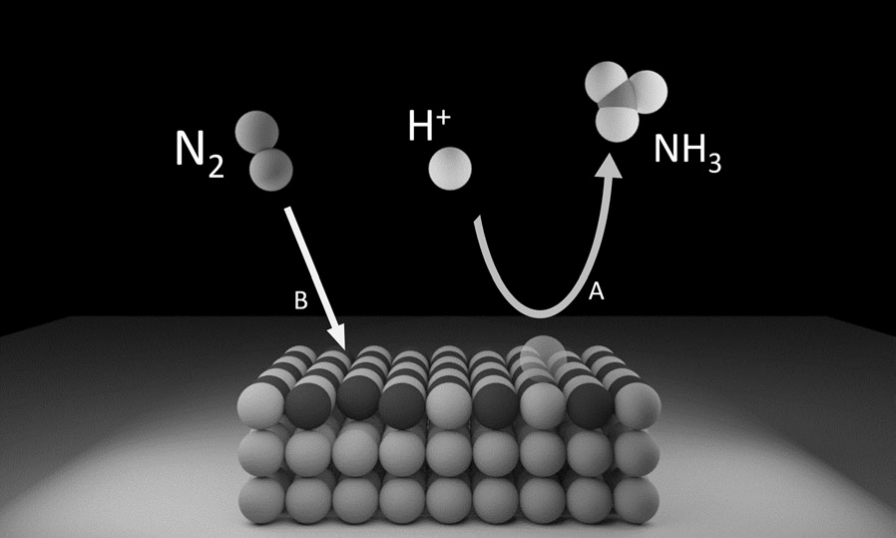
Schematic for Mars-van Krevelen reaction mechanism for N_2_ reduction to NH_3_ on the surface of transition metal nitrides (Reaction A: surface of metal nitride reduced to NH_3_, Reaction B: Refilling of the vacancy by gaseous N_2_)

The proposed reaction mechanism on a heterogeneous catalyst for the electrochemical N_2_ reduction to NH_3_ is classified into (1) *associative pathway* and (2) *dissociative pathway* [24]. In *associative pathway,* the N_2_ molecule binds to the catalyst surface and further undergoes hydrogenation with the two nitrogen centers bound to each other, and the NH_3_ molecule is released after the final N≡N is broken. *The associative pathway* can be subdivided further into two categories, the *distal pathway* and *alternating pathway* based on different hydrogenation sequences. In *distal pathway* the NH_3_ molecule is released after the remote N atom is hydrogenated first, and the hydrogenation process continues further to produce the other NH_3_ molecule, while in the *alternating pathway*, the two N atoms are hydrogenated synchronously as shown in Fig. 1 [24]. In the dissociative pathway, the N≡N bond is broken much before hydrogenation; thus, leaving two adsorbed N-atoms on the catalyst surface. Finally, the adsorbed N atom undergoes independent hydrogenation before conversion to NH_3_. Recently, Abghoui and Skúlasson proposed a possible reaction mechanism for N_2_ reduction to NH_3_ via Mars-van Krevelen (Mvk) as shown in the Scheme 1, which provides a much favorable reaction mechanism than compared to typical dissociative and associative mechanisms on the surface of transition metal nitride’s (TMN) [25]. In the Mvk mechanism for N_2_ reduction to NH_3_ on TMN, the N atom is reduced to NH_3_, and the catalyst regenerated through the gaseous N_2_ while differing from the conventional associative and dissociative mechanism. Further, density functional theory (DFT) calculations revealed that the dissociative mechanism hinders the dissociation of N_2_ on the clean surfaces of TMNs is endothermic while the activation barriers being large. The overpotential for N_2_ reduction to NH_3_ is predicted to be much smaller via the MvK mechanism than in the case of the associative mechanism [26, 27].

### Principles for designing NRR electrocatalysts

In electrochemical NRR usually, the formation of protons (H^+^) occurs at the anode/electrolyte interface. The pivotal step in NRR is the N binding to the catalyst surface and considering this for an ideal electrode material should have an optimized N binding. Various studies conducted on electrochemical NRR on transition metal under ambient conditions report the importance of N binding [28] A noteworthy point is that with weak binding the catalyst will be unable to activate the reactant, whereas when the binding is firm, there is a possibility that the catalyst being poisoned with the strongly adsorbed intermediates and in turn, creates a volcano-like trend between the bond strength and catalytic activity [16].

**Fig. 2:**
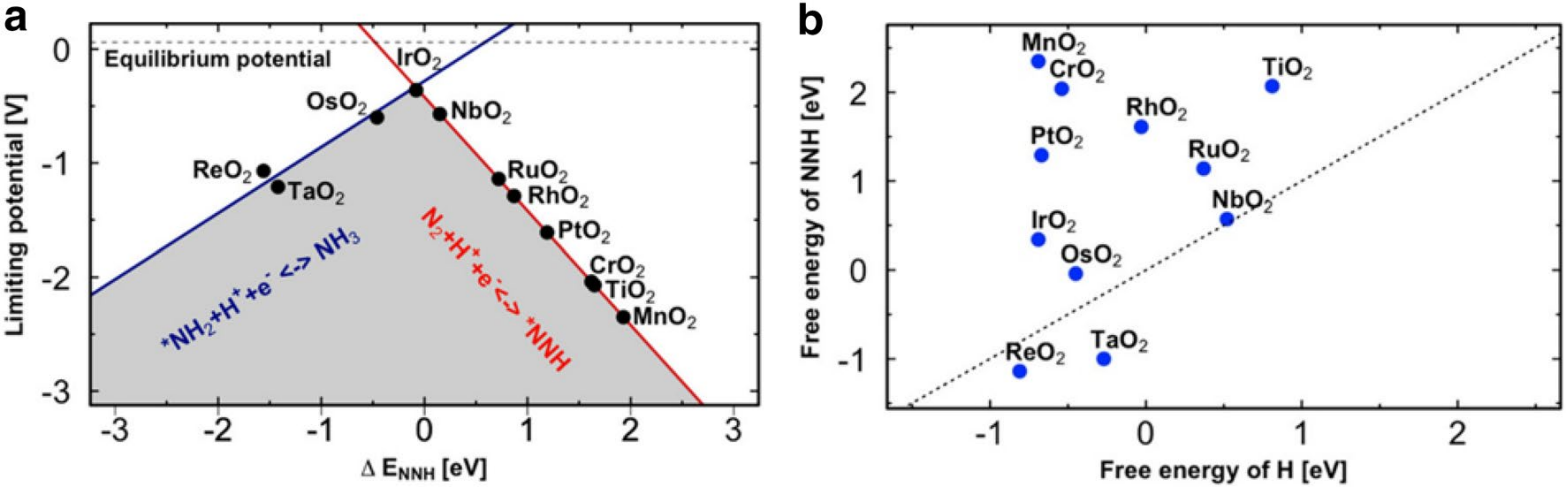
**a** Potential determining steps for the electrochemical N_2_ fixation to NH_3_ on metal oxide surfaces with binding energies of NNH. **b** Comparison of the free energy adsorption of NNH and that of H on metal oxide surfaces. (The dashed lines indicate where these free energies are equal. The oxides below these dashed lines show that NH_3_ formation can occur without being poisoned with protons) (Reproduced with permission [30] *Copyright 2017, American Chemical Society*)

These “volcano” diagrams, as displayed in Fig. 2 predict that metals such as Mo, Fe, Rh, and Ru are the most suitable catalyst for NRR activity [16]. However, at the same time, the HER volcano plot also summarizes that these metals are more active for HER than NRR at the same potentials, which reduces the Faradaic efficiency for the NRR while HER is the competing reaction. From the volcano plot, it was found that Mo and Fe to be the most promising candidates for electrochemical N_2_ reduction to NH_3_ via the associative mechanism. However, another issue is that these active sites on the catalyst surface could be occupied by oxygen rather than N_2_ in the presence of water thereby minimizing the efficiency of these catalysts. A recent computational study conducted on Mo nanoclusters shows that the active sites specifically bind oxygen over N_2_ and H_2_ [29]. It is therefore imperative that much negative potential is required to reduce the oxygen adsorption on the surface and promote N_2_ adsorption. The studies conducted for NRR on the surfaces of Ag and Cu with weak N binding showed that it was limited by the adsorption of N_2_ as *N_2_H as the first step and protonation of *NH to *NH_2_. However, in case of metals like Re that bind strongly to nitrogen, the removal of *NH_2_ to release NH_3_ is the limiting step [27]. In the case of noble metals like Pt [16] it has been found that the N-coverage is smaller when compared to H-adatoms at the same onset potential for NH_3_ formation, which is indicative of the fact that the HER is the most competing reaction with NRR.

The possibility of N_2_ reduction for NH_3_ activation under ambient conditions have been recently explored intensively on the (110) facet of several metal oxides [30], of them the most promising candidates turned out to be NbO_2_, ReO_2_, and TaO_2_. Metal nitrides are another class of highly researched materials that show promising results than its metal counterpart towards N_2_ reduction to NH_3_ than the competing HER. A DFT study performed by Abghoui and Skúlasson on TMN surfaces concluded by stating that a suitable catalyst should a have high reactivity and stability towards NRR and studies conducted on the surfaces of the mononitrides with Zincblende (ZB) and rocksalt (RS) structures shows that VN, CrN, NbN, ZrN having a rocksalt structure showed lower onset potential for NRR [27].

Among these polycrystalline VN exhibited lower overpotential (− 0.5 V) and prevented both HER and catalyst decomposition [27] 2D materials have also been researched, but these have not been employed much towards NRR. Recently, Azofra et al. [31] implied that 2D transition metal carbides such as MXenes are promising towards N_2_ reduction to NH_3_ based on DFT calculations. The N_2_ chemisorbed on the MXene nanosheets can elongate and weaken the N–N triple bond thereby promoting NRR. Despite these, the critical point to consider while designing an electrocatalyst for efficient NRR is the right material composition, crystal structure, textural structure (porosity), good electrical conductivity to promote electron transfer, and surface properties which would promote strong affinity towards N-adatoms binding rather than H-adatoms.

### Electrocatalysts for N_2_ fixation to ammonia

Electrocatalysts designed for N_2_ fixation to NH_3_ have been on the rise, and these catalysts could be categorized based on their ability to reduce N_2_ to NH_3_ under ambient conditions. Here we would like to discuss a few emerging electrocatalysts for NRR. In a recent study, Cui et al. demonstrated that both the NH_3_ yield and Faradaic efficiency could be improved by surface modification of hematite nanostructure, with a proof-of-concept wherein hematite electrocatalysts were fabricated with varying concentration of oxygen vacancies (OVs) while annealing in Argon (Ar) and air to modify OV concentration. They were able to achieve better catalytic performance with a higher concentration of OVs. Their *o*-Fe_2_O_3_-Ar/CNT catalyst produced a maximum NH_3_ production rate of 0.46 μg h^−1^ cm^−2^ at − 0.9 V vs. Ag/AgCl in 0.1 M KOH electrolyte with a Faradaic efficiency of 6.0%. The durability of the system was further evaluated and was surprisingly able to achieve a high NH_3_ production rate of 1.45 μg h^−1^ cm^−2^ with a Faradaic efficiency of 8.28% respectively. This work further elucidates the importance of active sites (oxygen vacancy defects) for N_2_ adsorption and activation. Carbon-based materials have received widespread attention in recent years owing to the natural abundance and feasibility in preparation methods. Recently Liu et al. [32] reported the N-doped porous carbon as an efficient electrocatalyst for N_2_ fixation to NH_3_. N-doped porous carbon showed an enhanced electrocatalytic activity with a high NH_3_ production rate of 1.40 mmol g^−1^ h^−1^ (− 0.9 V) with a maximum current efficiency of 1.42%. The authors believe that the high content of the pyridinic and pyrrolic N might be responsible for promoting the NH_3_ formation. DFT studies performed indicate that the preferred pathway for NH_3_ formation was through *N≡N→*NH=NH→*NH_2_–NH_2_→2NH_3_. The current study opens up the possibilities of exploring carbon-based materials for NRR under ambient conditions.

Recently Zhang et al. reported MoS_2_ as a proof-of-concept for NRR which was theoretically and experimentally confirmed to be active for NRR under ambient conditions. Density functional theory (DFT) calculations performed to study if the MoS_2_ edge sites are electrocatalytically active for NRR as it is well known that these edge sites are active for HER. The DFT calculations further performed suggests that the positively charged Mo-edge plays a vital role in polarizing and activating the N_2_ molecules. The potential-determining step (PDS) happens to be the reductive protonation of adsorbed N_2_, with a barrier potential of 0.68 eV without any externally applied potential. This relatively low barrier in terms of PDS on Mo-edge further strengthens the claim that MoS_2_ as a potential candidate for NRR. Appreciable NH_3_ yield of 8.08 × 10^−11^ mol s^−1^ cm^−2^ with a faradaic efficiency of 1.17% respectively. The MoS_2_ catalyst exhibited inferior NRR selectivity under acidic conditions than in alkaline conditions, as the electrolyte is infested mostly with protons which would ultimately result in hydrogen evolution. This study offers an exciting new avenue to explore transition metal sulfides as an attractive NRR electrocatalyst for N_2_ reduction to NH_3_ [33].

Transition metal nitrides-based electrocatalysts have gathered tremendous as they offer an added advantage for the presence of N-adatoms in the crystal structure of the metal atom. Zhang et al. [34] reported the synthesis of MoN nanosheet array as a promising NRR electrocatalyst. They were able to achieve an NH_3_ production rate of 3.01 × 10^−10^ mol s^−1^ cm^−2^ with a Faradaic efficiency of 1.15%. Further DFT calculations proposed that the MoN nanoarray catalyzes NRR via MvK mechanism. This study opened new doors for the rational design of MoN nanocatalysts for efficient NRR.

Another essential breakthrough for the use of TMN catalysts in electrocatalytic NRR recently was proposed by Yang et al. based on Vanadium Nitride nanoparticles as an electrocatalyst for NRR under ambient conditions. They were able to achieve a high NH_3_ yield rate of 3.3 × 10^−10^ mol s^−1^ cm^−2^ with high faradaic efficiency of 6.0% (− 0.1 V) in the very first hour of NRR studies. A steady rate of NH_3_ production was reported with a yield rate of 1.1 × 10^−10^ mol s^−1^ cm^−2^ and a Faradaic efficiency of 1.6% even after 116 h. Further studies conducted provided elucidative results that the electrochemical NRR proceeded via MvK mechanism. This was supported through ^15^N_2_ isotope tests. Combined with the ex situ and operando studies that the surface of VN_0.7_O_0.45_ is the active phase for electrochemical NRR and the conversion to VN would result in deactivation of the catalyst. This hypothesis is supported also by the DFT studies performed [35].

Song et al. reported N-doped Carbon Nanospikes (CNS) as an efficient electrocatalyst for efficient N_2_ fixation to NH_3_. The proposed mechanism for the reaction depends on the physical interactions on the sharp surface of the catalyst in the absence of a transition metal on CNS. This mechanism was supported by a control experiment performed with an O-etched CNS where the blunt tips produced small fractions of NH_3_ under the same conditions. They were able to achieve a maximum yield of 97.18 μg h^−1^ cm^−2^ with high Faradaic efficiency of 11.56%. The study also stressed on the importance of counterions in the aqueous electrolyte in enhancing the NH_3_ production rates in the order of Li^+^ > Na^+^>K^+^ wherein these small counterions increased the N_2_ concentration within the Stern layer. H_2_ evolution was suppressed with the formation of the dehydrated cation layer surrounding the tip while minimizing the access to water molecules and allowing access to N_2_ molecules in a high electric field. Although this theory requires further elucidation to fully understand this reaction mechanism, which includes the energy needed for the injection of N_2_ and the solvation of N_2_ and both the counterions. This study provides a viable physical reaction mechanism for electrolysis of N_2_ to NH_3_ [36].

Nanoscale confinement is a crucial aspect of improving N_2_ selectivity on the electrocatalysts surface. Recently Nazemi et al. reported the electrosynthesis of NH_3_ from N_2_ and water under ambient conditions with hollow gold nanocages (AUHNCs) as the catalyst. The significant findings from this study are the interdependency of the Ag content in the interior of the Au hollow nanoparticles, pore size/density and finally, the total surface area of the nanoparticles contributed for the enhanced electrocatalytic activity for NRR. Another important finding was the presence of Ag in the cavity of AuHNCs-635 was found to decrease the electrocatalytic activity towards NRR as Ag enhances H_2_ evolution. Among the various shapes tested for NRR AuHNCs with Localized surface plasmon resonance (LSPR) value of 715 showcased highest NH_3_ yield rates of 3.74 μg cm^−2^ h^−1^ and high Faradaic efficiency of 35.9% was achieved at − 0.4 V vs. RHE. The higher FE and NH_3_ yield rates of AuHNCs-715 resulted from the better selectivity for N_2_ reduction as the pore size, Ag content in the cavity and the active surface area of the nanoparticle played a crucial role in enhancing N_2_ selectivity. As primarily NRR happens in the cavities of AuHNCs, a smaller pore didn’t enhance the N_2_ selectivity as a higher Ag content resulted in lower selectivity with increased H_2_ evolution. Further increase in pore size results in decreased NRR selectivity due to the decreased surface area and lack of confinement of the reactants within the cavities (AuHNCs-795). Compared to AuHNCs with larger pore size but do not have Ag in the cavities. Further increase in pore size results in decreased NRR selectivity due to the decreased surface area and lack of confinement of the reactants within the cavities (AuHNCs-795) [37].

Recently Yao et al. reported the use of Chromium Oxynitride (CrO_0.66_N_0.56_) as an efficient electrocatalyst for N_2_ fixation to NH_3_ using a homemade proton exchange membrane electrolyzer (PEMEL) under ambient conditions. A high NH_3_ yield rate of 8.9 × 10^−11^ mol s^−1^ cm^−2^ with high Faradaic efficiency of 6.7% was achieved at 2.0 and 1.8 V respectively. The NRR activity of CrO_0.66_N_0.56_ is better than pure Cr_2_O_3_ and CrN catalysts. The study also provided more insights into the improved electronic properties of metal nitrides through partial oxidation which opens the door towards designing rational electrocatalysts for NRR [38].

## Reaction mechanism for photocatalytic NRR

Photocatalytic N_2_ reduction to NH_3_ typically follows several steps before the release of the NH_3_ molecule. The first step involves the occupation of the conduction band by the photogenerated electrons which leaves behind a vacancy in the valance band that will be occupied by holes. Subsequently, some these photogenerated electrons and holes recombine while some drift to the surface of the catalyst and take part in the redox reactions on the surface. At this point, H_2_O can be oxidized to O_2_ by the holes whereas N_2_ reduced to NH_3_ after a series of multiple generations of electrons and water-derived protons [39, 40].

A well-known fact regarding photocatalytic redox reaction is that it is entirely reliant on the reduction potential of the adsorbate and band position of the semiconductor electrode [41–43] For instance, it is necessary that the position of the conduction band of a semiconductor electrode should be higher (more negative) in relative to the reduction potential required for N_2_ hydrogenation, while the valance band position is located much lower (more positive) for oxygen evolution potential [44–46]. This leads to limitations which mainly revolves around two aspects one being the ability to active N_2_ molecule for NH_3_ formation. This also requires developing semiconductor electrodes with small bandgap preferably along the visible light region, which would still fulfill the necessary thermodynamic conditions for the reduction of N_2_ to NH_3_. The second limitation is the necessity to suppress the recombination of the charge carriers to obtain a higher solar conversion efficiency and apparent quantum yield for the reaction [46].

### Design principles for photocatalytic NRR

**Fig. 3:**
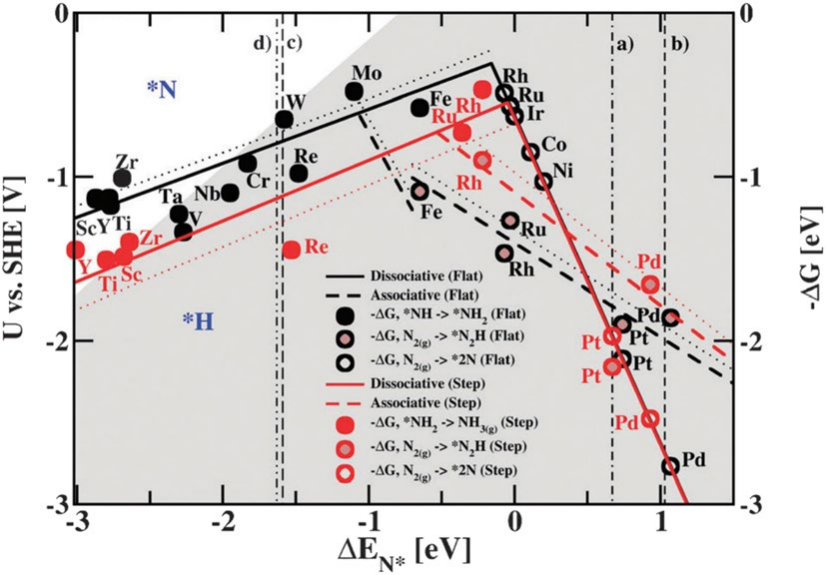
Volcano plot for the NRR on metal surfaces with specific mechanistic assumptions. A shaded area in the plot represents the overlaid volcano diagram for the HER counterpart (Reproduced with permission [16] *Copyright 2012, Royal Society of Chemistry*)

Several recent investigations carried out from the experimental and theoretical viewpoint on the N_2_ reduction mechanisms over various photocatalysts [31, 47, 48]. For a better understanding of the N_2_ reduction to NH_3_ especially on hydrogenation on photocatalysts it is necessary to understand the process in greater detail. For instance, in the first photogenerated electron transfer, the N_2_ adsorbed on the catalyst surface attains a proton (H +) from the environment and a photogenerated electron from the catalyst to generate the necessary chemical species. Photocatalytic N_2_ fixation differs from conventional N_2_ reduction on transition metals because the former is a chemical process occurring on a semiconductor surface. Which makes the entire catalytic activity becomes reliant on the surface chemistry of semiconductors. Electrocatalytic NRR can be realized through electricity obtained from solar cells and wind turbines but in the case of photocatalytic NRR which can utilize the renewable energy source (solar) for N_2_ fixation to NH_3_ (Fig. 3).

### Photocatalysts for N_2_ fixation to NH_3_

**Fig. 4:**
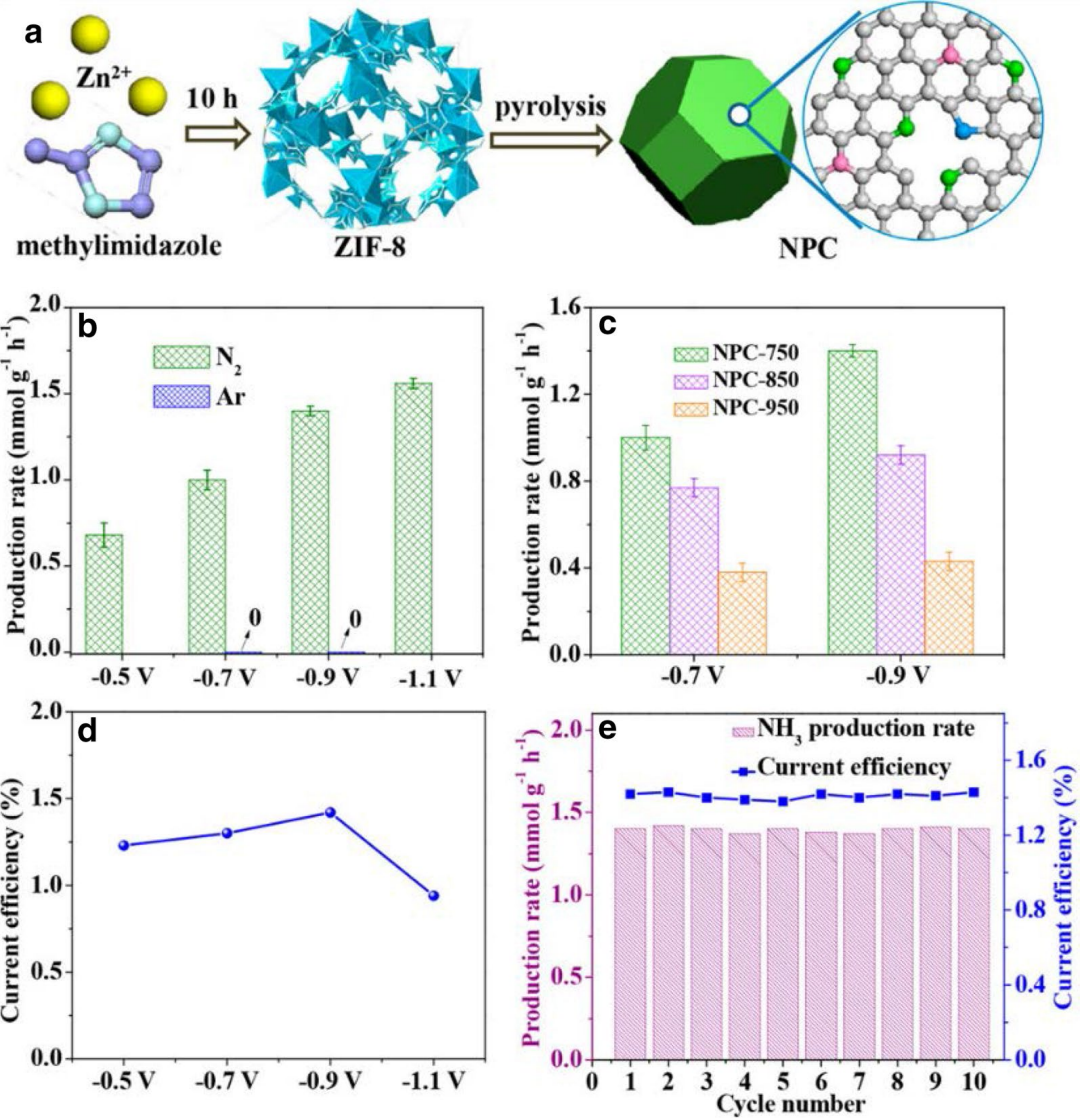
**a** Graphical illustration of the NPC preparation method. **b** NH_3_ production rates for NPC-750, NPC-850, and NPC-950. **c** NH_3_ production rates at − 0.74 V and − 0.9 V for NPC-750,850 and 950 **d** Current efficiency of NPC-750. **e** Recyclability test for NPC-750 for 10 consecutive cycles at − 0.9 V (Reproduced with permission [32] *Copyright 2017, American Chemical Society*)

**Fig. 5:**
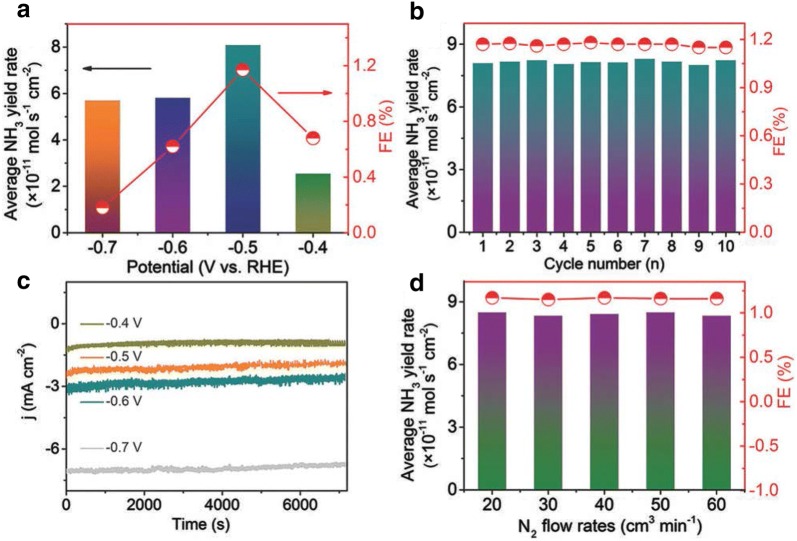
**a** Average NH_3_ yield and Faradaic efficiency of MoS_2_/CC at different potentials. **b** Recycling test performed at − 0.5 V. **c** Time-dependent current density curves for MoS_2_/CC for varying NRR potentials. **d** Yield ad Faradaic efficiency at different N_2_ flow rates at − 0.5 V (Reproduced with permission [33] *Copyright 2018, Wiley*–*VCH*)

**Fig. 6:**
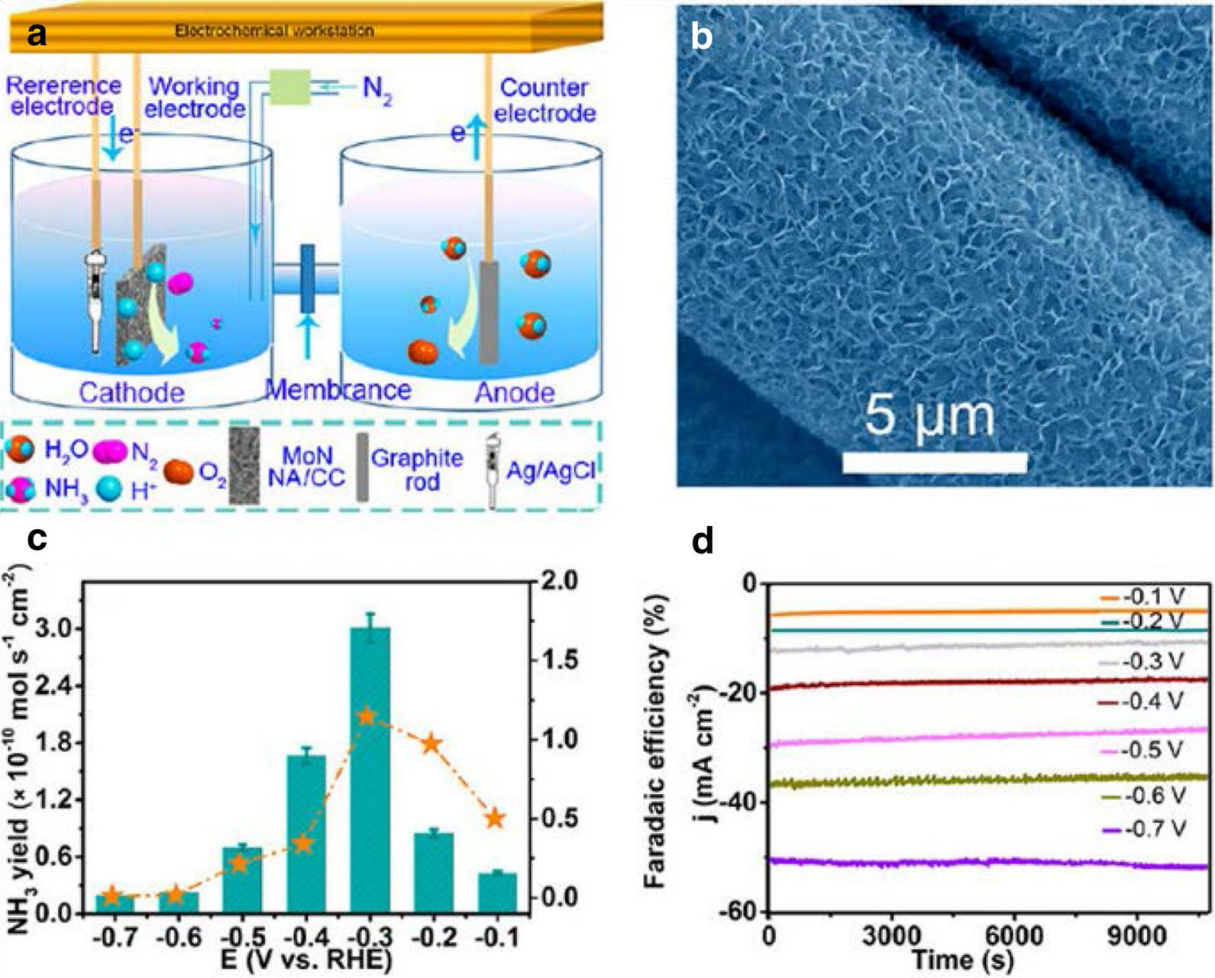
**a** Schematic illustration for electrocatalytic NRR. **b** SEM image showing MoN NA/CC. **c** NH_3_ yields and FE at different potentials. **d** Chronoamperometry results at various potentials (Reproduced with permission [34] *Copyright 2018, American Chemical Society*)

**Fig. 7:**
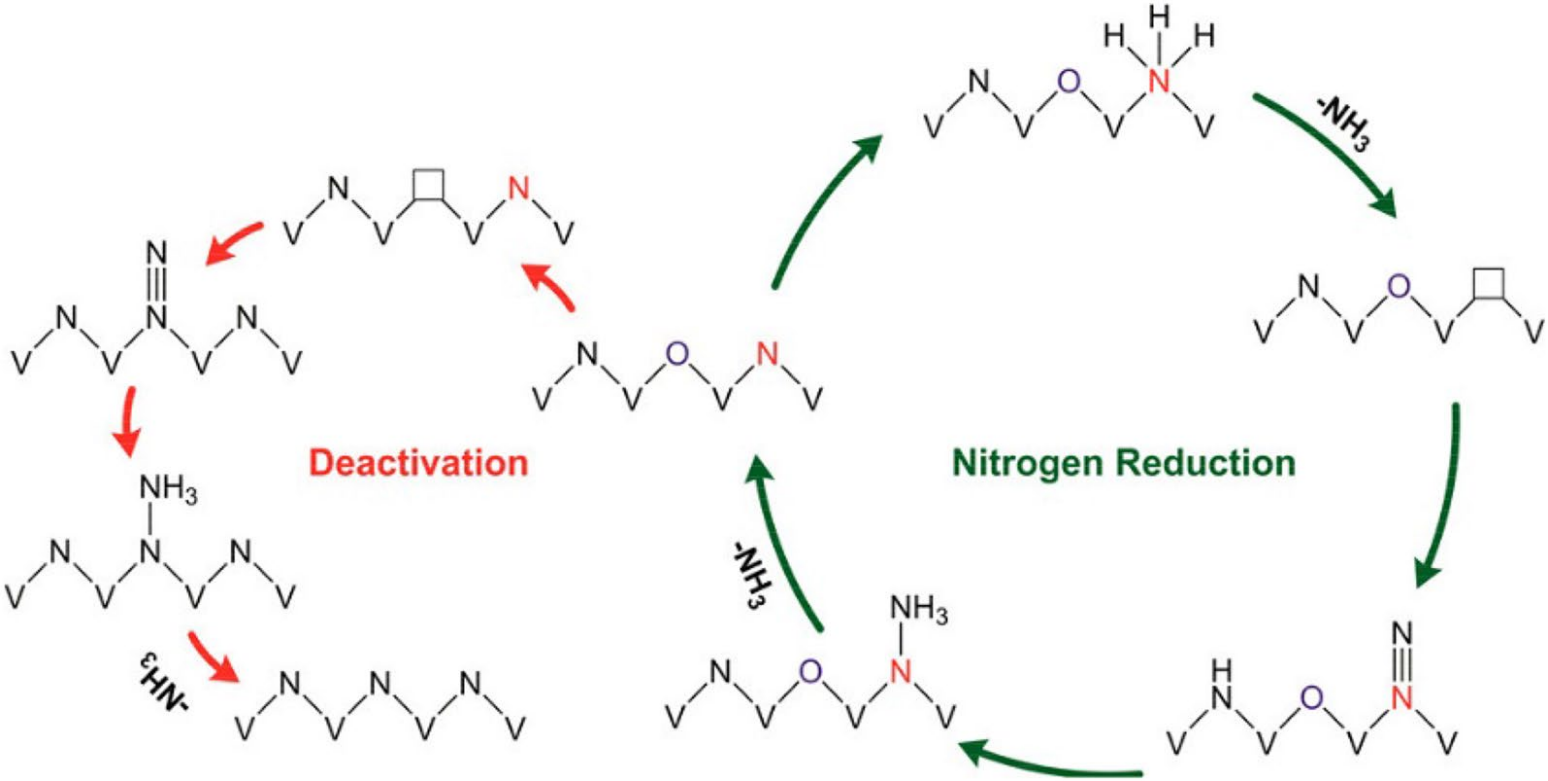
The proposed reaction pathway for N_2_ reduction on VN_0.7_O_0.45_ via MvK mechanism and the deactivation of the catalyst (Reproduced with permission [35] *Copyright 2018, American Chemical Society*)

**Fig. 8:**
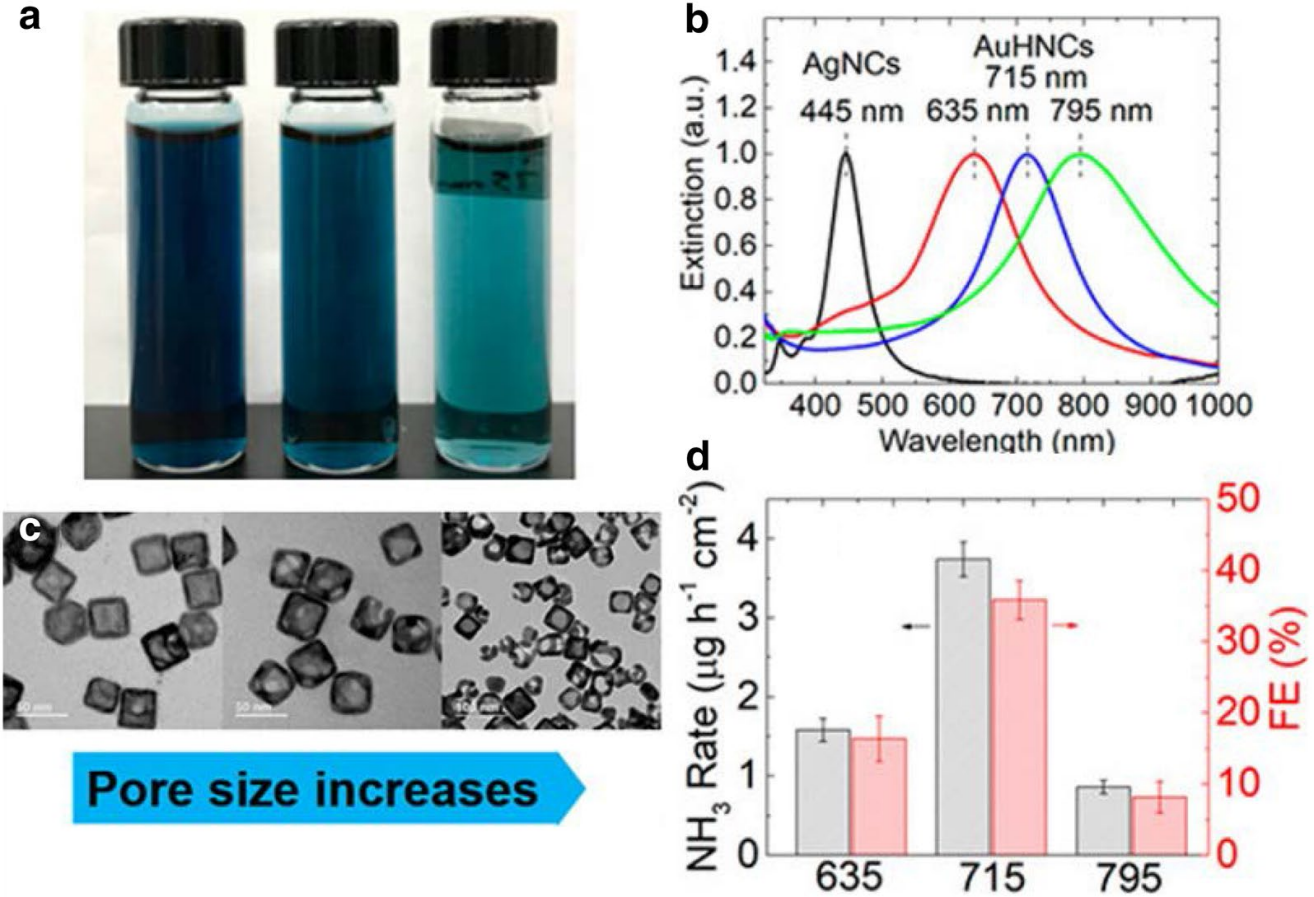
**a** AUHNCs dispersed in DI water. **b** UV–vis spectra of AgNCs and AuHNCs with various peak LSPR values. **c** TEM images displaying AuHNCs with peak LSPR at 635, 715 and 795 nm. **d** NH_3_ yield rate and Faradaic efficiency for the various LSPR peak values at − 0.4 V (Reproduced with permission [37] *Copyright 2018, American Chemical Society*)

**Fig. 9:**
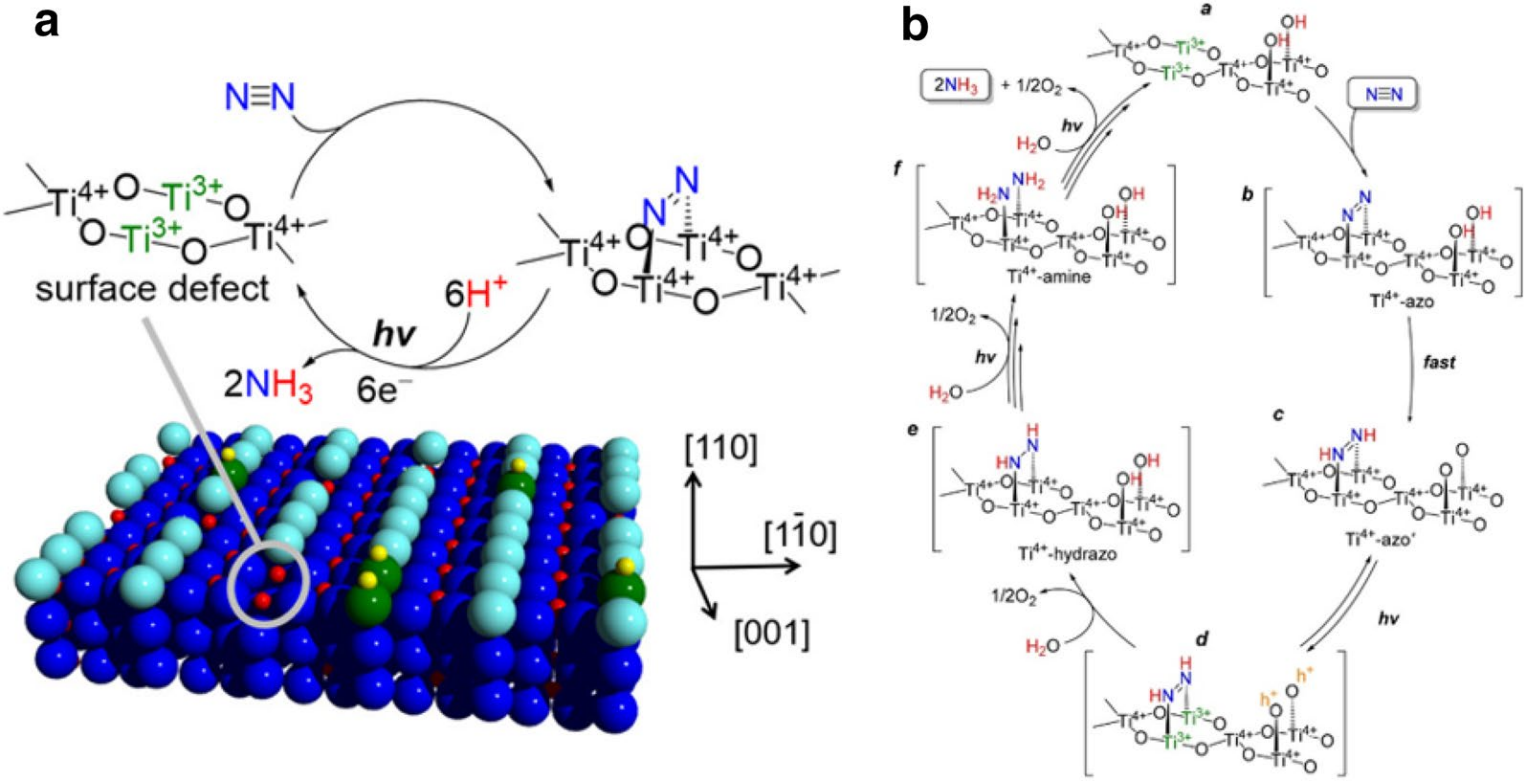
**a** Photocatalytic N_2_ fixation on the surface of Rutile TiO_2_ (110) surface. **b** The mechanism for the photocatalytic fixation of N_2_ to NH_3_ over the surface oxygen vacancies of TiO_2_ (Reproduced with permission [49] *Copyright 2017, American Chemical Society*)

**Fig. 10:**
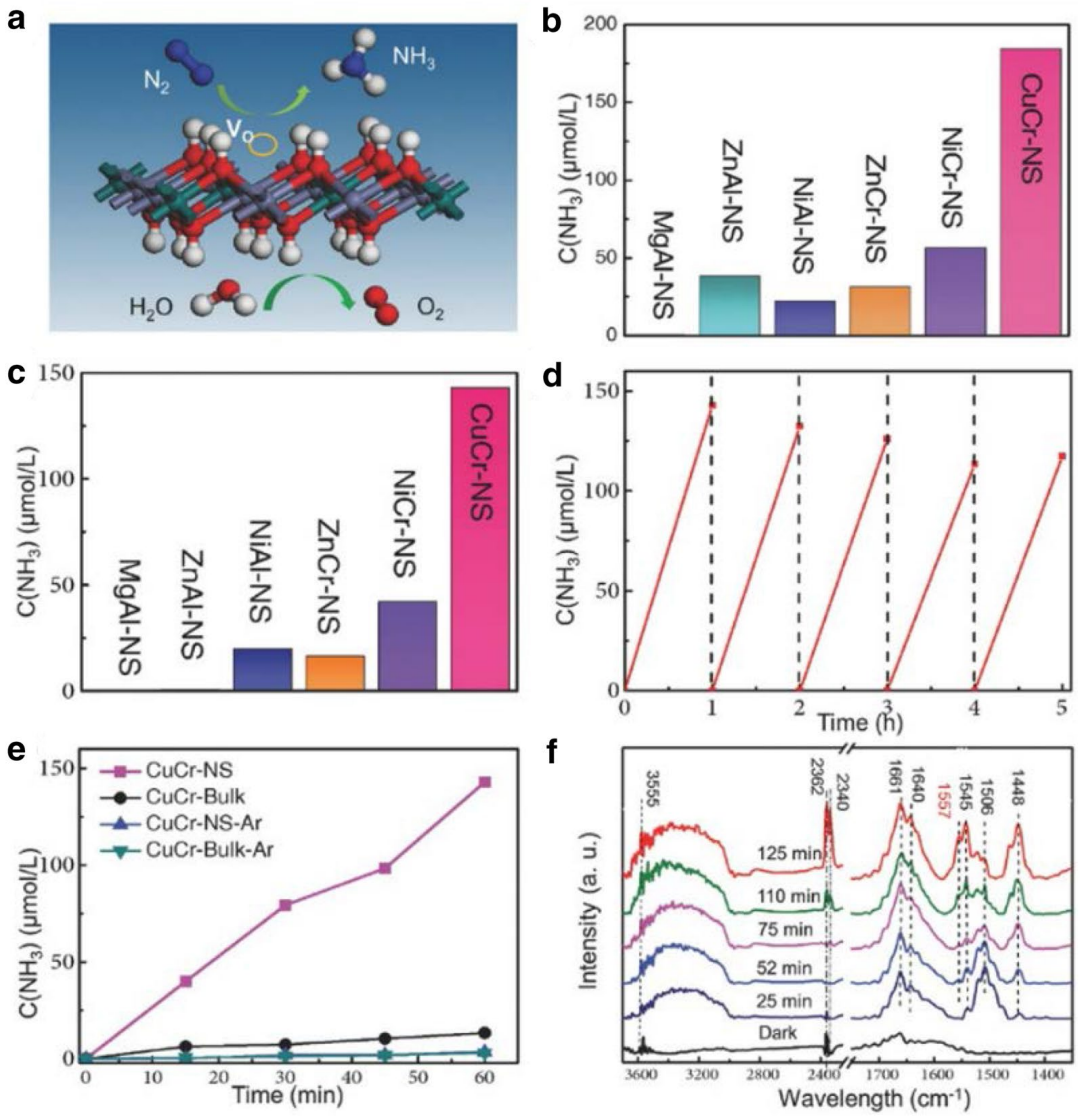
**a** Illustration of the N_2_ fixation process. **b**, **c** NH_3_ yield over a 1 h test period on different LDH photocatalysts with UV-vs illumination. **d** Catalyst stability tests performed for CuCr-NS in N_2_ under visible-light illumination. **e** Time-dependent NH_3_ evolution under N_2_ and Ar. **f** In situ IR spectra recorded for CuCr-NS under N_2_ during 125 min UV–vis illumination (Reproduced with permission [50] *Copyright 2017 Wiley*–*VCH*)

Recently N_2_ fixation to NH_3_ on TiO_2_ surface was studied with the catalytic conversion taking place on surface oxygen vacancies [49]. Various catalysts were tested among them JRC-TIO-6 (rutile phase) displayed highest N_2_ reduction activity, and it showcased an enhancement of 2.7 times in the catalytic activity when 2-PrOH used as a sacrificial e^−^ donor for the entire duration of the reaction (12 h). From Fig. 4 it can be seen that the superficial Ti^3+^ surface acts as an electron donor by providing numerous number of active sites for N_2_ reduction to NH_3_ which eases the process of N ≡ N bond dissociation. They were able to achieve an NH_3_ yield rate of 1.75 mmol h^−1^ with a solar-to-chemical conversion efficiency of 0.02% (Figs. 5, 6, 7, 8, 9 and 10).

Two-dimensional (2D) nanosheets represent important class photocatalysts among which layered double hydroxides (LDH) show promising results for NRR. Zhao et al. reported the synthesis of ultrathin LDH nanosheets of the type M^II^ M^III^ (where M^II^ = Mg, Zn, Ni, Cu; and M^III^ = Al, Cr) as a photocatalyst material for NRR. The NRR activity followed the trend of CuCr-NS (184.8 μmol L^−1^) > NiCr-NS (56.3 μmol L^−1^) > ZnCr-NS (31.2 μmol L^−1^) > ZnAl-NS (38.2 μmol L^−1^) > NiAl-NS (22.3 μmol L^−1^). Furthermore, MgAl-NS was found to be inactive which presumably is due to its larger bandgap (~ 5.0 eV). CuCr-NS showed better photocatalytic performance with a Faradaic efficiency of 0.44% and displayed excellent photocatalytic stability without any obvious decrease even after five consecutive cycles. The reason for the enhanced catalytic activity was the distortions in the MO_6_ octahedra caused by the incorporation of oxygen vacancy defects within the ultrathin LDH nanosheets. Density functional theory (DFT) studies indicate that the introduction of oxygen vacancy defects further introduces gap states that promote N_2_ adsorption and facilitate photoinduced charge transfer from LDH to N_2_, while at the same time serving as active sites for the chemical reaction of N_2_ and H_2_O to form NH_3_ and O_2_ respectively. The authors also compared the above results for different operating atmospheres of N_2_ and Ar. These LDH nanosheets with a large abundance of oxygen vacancies that enhanced the adsorption and activation of N_2_ with excellent photocatalytic activity in transforming N_2_ to NH_3_ under UV–vis excitation and in some selected cases visible excitation (CuCr-NS under wavelength > 500 nm). Further, the introduction of Cu (II) ions in the LDH nanosheets created additional structural distortions and compressive strains, thus leading to increased interaction between the LDH and N_2_, therefore promoting NH_3_ evolution. The study conducted here demonstrates a promising new strategy for reduction of N_2_ to NH_3_ using LDH nanosheets as photocatalysts with good NH_3_ yields under ambient conditions [50].

Dong et al. reported the synthesis of nitrogen vacancy (NV) incorporated g-C_3_N_4_ as a photocatalyst for NRR which was synthesized by annealing in nitrogen atmosphere. A high NH_3_ yield of 1240 μmol h^−1^ was obtained with g-C_3_N_4_ photocatalyst. It was believed that introducing nitrogen vacancies in g-C_3_N_4_ would generate N_2_ activation sites to enhance the photocatalytic activity towards NRR. These induced NV sites can selectively adsorb and activate N_2_ molecule. In addition to this NVs improved the separation efficiency of the photogenerated carriers and the electron transfer from g-C_3_N_4_ to the adsorbed N_2_. The study conducted here was the first of its kind to report how NVs affected the reactivity of semiconductors for photocatalytic N_2_ fixation [51].

Zhang et al. reported Mo-doped defect rich W_18_O_49_ ultrathin nanowires as efficient photocatalyst towards solar-driven nitrogen fixation. An NH_3_ yield of 195.5 μmol h^−1^ g^−1^ with a faradaic efficiency of 0.33% at 400 nm and a solar-to-NH_3_ efficiency of 0.028% was achieved under illumination from an AM 1.5G light irradiation in pure water. Further investigations revealed that Mo doping induces multi-synergetic effect on N_2_ activation and dissociation through the defect states in W_18_O_49_. Active sites were found to be the Mo-W centers for the chemisorption of N_2_ molecules whose heterogeneity polarizes the N_2_ molecule for better activation of the catalyst. The metal–oxygen covalency in the photocatalyst lattice promotes better electron transfer to N_2_ molecules. Mo-doping introduces an elevation of the defect band center towards the Fermi level which generates more energy into the photoexcited electrons for N_2_ fixation to NH_3_ [52].

Yang et al. reported nitrogen photofixation using plasmonic gold nanocrystal on ultrathin nanosheets. Based on the “working-in-tandem” concept of natural nitrogenase herein they have constructed a N_2_ photofixation system comprising of inorganic Au/TiO_2_-OV catalysts. The oxygen vacancies (OVs) acted as the activation sites for N_2_ molecules, whereas the plasmonic electrons acting as the reducing agents for the final fixation of the N_2_ to NH_3_. Au/TiO_2_-OV catalyst exhibited a high NH_3_ yield of 130.5 mmol h^−1^g^−1^ with an apparent quantum efficiency of 0.82% at 550 nm. The authors reported that the “working-in-tandem” strategy effectively tackles two important criteria for activation and reduction of N_2_ to form NH_3_. Thus, optimizing the absorption across the overall visible range with the mixture of Au nanospheres and nanorods which further elevates the N_2_ photofixation rate by 66.2% in comparison with the Au nanospheres solely [53] Tables 1 and 2 describes a brief summary on the representative NRR photocatalysts and electrocatalysts.

**Table 1:** A brief summary of the representative experimental studies on NRR using various electrocatalytic heterogeneous catalysts

Catalyst	Electrolyte	Condition	NH3 formation rate	Unit	Potential	Reference electrode	Faradaic efficiency	Year
Fe-phthalocyanine	1.0 M KOH	25 °C	7.0E+05	mol s^−1^ cm^−2^	− 47.8 mA cm^−2^	Current density	0.34%	1989 [59]
ZnS	1.0 M KOH	25 °C	7.1E+06	mol s^−1^ cm^−2^	− 0.1 V	vs RHE	0.964%	1990 [60]
ZnSe	1.0 M KOH	25 °C	8.1E+06	mol s^−1^ cm^−2^	− 0.1 V	vs RHE	1.29%	1990 [60]
Ti	0.2 M LiClO4/0.18 M ethanol in THF	25 °C			2.0 V	Cell Voltage	8.20%	1994 [60]
Cu	0.2 M LiClO4/0.18 M ethanol in THF	25 °C			2.0 V	Cell Voltage	5.30%	1994 [61]
Ru/C	2.0 M KOH	20 °C	3.43137E−12	mol s^−1^ cm^−2^	− 1.10 V	vs Ag/AgCl	0.28%	2000 [62]
		90 °C	4.08497E−12	mol s^−1^ cm^−2^	− 0.96 V	vs Ag/AgCl	0.92%	2000 [62]
Polyaniline	methanol/LiCiO_4_/H_2_SO_4_	25 °C	0.000,000,014	mol^−1^ ml^−1^	− 0.12 V	vs RHE	2.00%	2005 [63]
30 wt % Pt/C	0.50 M H_2_SO_4_	RT	1.14E−09	mol s^−1^ cm^−2^	1.6 V	Cell Voltage	0.50%	2013 [64]
	H^+^/Li^+^/NH_4_^+^ mixed electrolyte	80 °C	9.37E−10	mol s^−1^ cm^−2^	1.2 V	Cell Voltage	0.83%	2013 [65]
Ru/Ti	0.50 M H_2_SO_4_	30 °C	1.2E−10	mol s^−1^ cm^−2^	− 0.15 V	vs NHE	N/A	2014 [66]
Rh/Ti			1.5E−11	mol s^−1^ cm^−2^	− 0.171 V			
Ni wire	0.050 M H_2_SO_4_/0.1 M LiCl, EDA	RT	3.58E−11	mol s^−1^ cm^−2^	1.8 V	Cell Voltage	17.20%	2016 [58]
Porous Ni	2-propanol/H_2_SO_4_	RT	1.54E−11	mol s^−1^ cm^−2^	0.5 mA cm^−2^	Current Density	0.89%	2016 [67]
Mo nanofilm	0.010 M H_2_SO_4_	RT	3.09E−11	mol s^−1^ cm^−2^	− 0.49 V	vs RHE	0.72%(at − 0.29 V vs RHE)	2017 [54]
γ-Fe_2_O_3_	0.10 M KOH	65 °C	1.21528E−11	mol s^−1^ cm^−2^	0 V	vs RHE	1.96%	2017 [68]
Au nanorods	0.10 M KOH	RT	2.69281E−11	mol s^−1^ cm^−2^	− 0.2 V	vs RHE	4.00%	2017 [55]
Au/TiO2	0.10 M HCl	RT	3.49673E−10	mol s^−1^ mg_cat_^−1^	− 0.2 V	vs RHE	8.11%	2017 [56]
Au-CeOx/RGO	0.10 M KOH	RT	1.35621E−10	mol s^−1^ mg_cat_^−1^	− 0.2 V	vs RHE	10.10%	2017 [57]
30 wt % Fe_2_O_3_-CNT	0.50 M KOH	RT	6.74E−12	mol s^−1^ cm^−2^	− 2.0 V	vs Ag/AgCl	0.16%	2017 [69]
PEBCD (poly *N*-ethyl-benzene-1,2,4,5-tetracarboxylic diimide)/C	0.50 M Li_2_SO_4_	25 °C	2.5817E−11	mol s^−1^ cm^−2^	− 0.5 V	vs RHE	2.85%	2017 [70]
		40 °C	7.07516E−11	mol s^−1^ cm^−2^	− 0.5 V		4.87%	
MOF(Fe)(metal–organic-frameworks)	2.0 M KOH	90 °C	2.12E−09	mol s^−1^ cm^−2^	1.2 V	Cell Voltage	1.43%	2017 [71]
Fe_2_O_3_-CNT	2.0 M NaHCO_3_	RT	3.59477E−12	mol s^−1^ cm^−2^	− 2.0 V	vs Ag/AgCl	0.15%(at − 1.0 V vs Ag/AgCl)	2017 [72]
Fe on stainless steel mesh	Ionic liquid	RT	3.88889E−10	mol s^−1^ cm^−2^	− 0.8 V	vs NHE	30%	2017 [73]
N-doped carbon	0.05 M H_2_SO_4_	RT	3.88889E−16	mol s^−1^ mg^−1^	− 0.9 V	vs RHE	1.42%	2018 [32]
Ru nanosheets	0.10 M KOH	RT	3.90196E−10	mol s^−1^ mg_cat_^−1^	− 0.2 V	vs RHE	0.217%	2018 [74]
Mo_2_N nanorod	0.1 M HCl	25 °C	1.28105E−09	mol s^−1^ mg_cat_^−1^	− 0.3 V	vs RHE	4.50%	2018 [75]
VN nanowire array	0.1 M HCl	25 °C	2.48E−10	mol s^−1^ cm^−2^	− 0.3 V	vs RHE	3.58%	2018 [76]
Pt	6 M KOH/polymer gel	30 °C	4.049E−11	mol s^−1^ cm^−2^	0.5 V	Cell Voltage	0.0108%	2018 [77]
Ir		60 °C	2.763E−11	mol s^−1^ cm^−2^	0.25 V	Cell Voltage	0.108%	
Bi_4_V_2_O_11_/CeOx	HCl, PH = 1	RT	3.79248E−10	mol s^−1^ mg_cat_^−1^	− 0.2 V	vs RHE	10.16%	2018 [78]
Pore-size-controlled hollow gold nanocatalysts	0.1 M LiOH	RT	6.11111E−11	mol s^−1^ cm^−2^	− 0.4 V	vs RHE	35.90%	2018 [37]
Hollow gold nanocages	0.5 M LiClO_4_	RT	6.37255E−11	mol s^−1^ cm^−2^ at − 0.5 V	− 0.4 V	vs RHE	30.20%	2018 [79]
MoN Nanosheets	0.1 M HCl	RT	3.01	mol s^−1^ cm^−2^	− 0.3 V	vs RHE	1.15%	2018 [34]
N-doped carbon nanospikes	0.25 M LiClO_4_	RT	1.58791E−09	mol s^−1^ cm^−2^	− 1.19 V	vs RHE	11.56 ± 0.85%	2018 [36]
Vanadium Nitride Nanoparticles	3 M KOH	RT	3.31E−10	mol s^−1^ cm^−2^		vs RHE	5.95%	2018 [35]
Chromium Oxynitride nanoparticles	Nafion Solution(5% wt)	RT	8.9E−11	mol s^−1^ cm^−2^	2.0 V	Cell Voltage	6.70%	2018 [38]
MoS_2_	0.1 M Na_2_SO_4_	RT	8.08E−11	mol s^−1^ cm^−2^	− 0.5 V	vs RHE	1.17%	2018 [33]

**Table 2:** A brief summary of the representative experimental studies on NRR using various photocatalytic heterogeneous catalysts

Catalyst	Electrolyte	Condition	NH3 formation rate	Unit	Quantum efficiency	Light source	Year
FeMoS-chalcogels	Pyridinium hydrochloride and sodium ascorbate	25 °C	4.44E−11	mol s^−1^	N/A	150 W Xenon Lamp	2015 [20]
BiOBr nanosheets	Water	25 °C	2.89E−08	mol s^−1^ g^−1^	0.23%	Visible Light (λ > 420 nm)	2015 [21]
Graphitic carbon nitride(g-C_3_N_4_)	0.1 M Na_2_SO_4_	RT	3.44444E−07	mol s^−1^ g^−1^	N/A	300 W Xe lamp (λ > 420 nm)	2015 [51]
CdS:nitrogenase MoFe protein biohybrid	0.5 M HEPES	25 °C	5.25E−06	mol s^−1^ g^−1^	3.30%	~3.5 mW cm^−2^ of 405 nm	2016 [19]
Titanium dioxide	Water	RT	1.01273E−09	mol s^−1^ g^−1^	0.02% (Solar to chemical energy)	1sun	2017 [49]
CuCr-LDH(Layered-Double-Hydroxide) Nanosheets	1 M KOH	25 °C	1.85E−04	mol L^−1^	N/A	300 W Xe lamp (λ > 400 nm)	2017 [50]
Bi_2_WO_6_ by cyclized polyacrylonitrile (c-PAN)	Water	RT	3.89E−08	mol s^−1^ g^−1^	N/A	visible light provided by a 300 W Xe lamp	2018 [80]
Mo-doped W_18_O_49_ ultrathin nanowires	0.5 M Na_2_SO_4_	RT	5.43E−08	mol s^−1^ g_cat_^−1^	0.33%	300 W Xe lamp	2018 [52]
Au nanocrystal-decorated ultrathin TiO_2_ nanosheets	Water and Methanol		3.63E−08	mol s^−1^ g^−1^	0.82%	300 W Xe lamp (λ > 420 nm)	2018 [53]
ECG (electrochemical grade) boron-doped diamond	Water	25 °C	5.55556E−11	mol s^−1^	N/A	450 W high-pressure Hg/Xe lamp (λ > 180 nm)	2013 [22]
Plasmon-enhanced black silicon	SO_3_^2−^ Solution	25 °C	2.12418E−09	mol s^−1^ cm^−2^	0.003%	2suns	2018 [23]

## Strategies for improving selectivity towards N_2_ fixation to NH_3_

Current systems for NRR lack efficient electro/photocatalyst for N_2_ reduction to NH_3_. Most of the catalysts reported so far are limited in terms of the large overpotential required for N_2_ reduction to NH_3_ and the low faradaic efficiencies of these systems are major concerns. The thermodynamics of NRR suggest that the reaction should proceed towards negative potentials wherein HER is more influenced this raises the question of selectivity. The other factors that affect N_2_ reduction to NH_3_ lies in the electrode material, conductivity of the catalyst material, electrolyte used, operating conditions (temperature), and applied potential.

The intrinsic reactivity can be enhanced by incorporating various strategies which include effects of crystal facets, [54, 55] size effects, [56] and amorphous nature of the catalyst, [57] have been found to be very decisive for electro/photocatalytic NRR. As discussed earlier limiting the availability of protons or electrons at the surface of the catalysts is another important aspect to suppress HER and provide selectivity towards NRR. Many strategies have been explored and recently to limit the concentration in the solution researchers have made use of mixture of electrolytes for aqueous based systems [58]. It is also very important to validate that the origin of NH_3_ produced from N_2_ is appropriately quantified using experiments such as ^15^N_2_ reduction and measured using ^1^H-NMR. Finally, the electron transfer rate at the interface between the current collector and active material and the interface between the electrode and electrolyte can also play an important role in minimizing HER and showing selectivity towards NRR. One must also take into consideration the underlying drawbacks that needs to be dealt while trying to limit the electron transfer rate towards HER as would result in lesser faradaic efficiencies of the system. It would be therefore astute to have an optimum balance between selectivity to attain a higher performance.

## Conclusion

In summary, we have discussed the advancement in electro/photocatalytic NRR and the underlying theoretical and experimental progress so far. Despite all the challenges and obstacles ahead we believe electro/photocatalytic NRR is much more realizable with its current growth. Moreover, in the near future, theoretical and experimental advancement coupled with operando/in situ studies will further shift the tide in developing much efficient electro/photocatalysts which much higher selectivity towards N_2_ reduction for NH_3_ formation.

## References

[1] Galloway JN, Townsend AR, Erisman JW, Bekunda M, Cai Z, Freney JR, Martinelli LA, Seitzinger SP, Sutton MA (2008). Science.

[2] Appl, M.J.F. NH3 principles and industrial practice. **245**, 12 (1999)

[3] Liu H (2014). Chin. J. Catal..

[4] Van der Ham CJ, Koper MT, Hetterscheid DG (2014). Chem. Soc. Rev..

[5] Tanabe Y, Nishibayashi Y (2013). Coord. Chem. Rev..

[6] Bratsch SG (1989). J. Phys. Chem. Ref. Data.

[7] Lan R, Irvine JT, Tao S (2012). Int. J. Hydrogen Energy.

[8] Christensen CH, Johannessen T, Sørensen RZ, Nørskov JK (2006). Catal. Today.

[9] Sun K, Moreno-Hernandez IA, Schmidt WC, Zhou X, Crompton JC, Liu R, Saadi FH, Chen Y, Papadantonakis KM, Lewis NS (2017). Energy Environ. Sci..

[10] RF Service (2014). Science.

[11] Oshikiri T, Ueno K, Misawa H (2016). Angew. Chem..

[12] Jia HP, Quadrelli EA (2014). Chem. Soc. Rev..

[13] Zhu YP, Guo C, Zheng Y, Qiao SZ (2017). Acc. Chem. Res..

[14] Yue M, Wang R, Cheng N, Cong R, Gao W, Yang T (2016). Sci. Rep..

[15] Duman LM, Farrell WS, Zavalij PY, Sita LR (2016). J. Am. Chem. Soc..

[16] Skulason E, Bligaard T, Gudmundsdóttir S, Studt F, Rossmeisl J, Abild-Pedersen F, Vegge T, Jonsson H, Nørskov JK (2012). Phys. Chem. Chem. Phys..

[17] Fujishima A, Honda K (1972). Nature.

[18] Schrauzer GN, Guth TD (2002). J. Am. Chem. Soc..

[19] Brown KA, Harris DF, Wilker MB, Rasmussen A, Khadka N, Hamby H, Keable S, Dukovic G, Peters JW, Seefeldt LC, King PW (2016). Science.

[20] Banerjee A, Yuhas BD, Margulies EA, Zhang Y, Shim Y, Wasielewski MR, Kanatzidis MG (2015). J. Am. Chem. Soc..

[21] Li H, Shang J, Ai Z, Zhang L (2015). J. Am. Chem. Soc..

[22] Zhu D, Zhang L, Ruther RE, Hamers RJ (2013). Nat. Mater..

[23] Ali M, Zhou F, Chen K, Kotzur C, Xiao C, Bourgeois L, Zhang X, MacFarlane DR (2016). Nat. Commun..

[24] Shipman MA, Symes MD (2017). Catal. Today.

[25] Abghoui Y, Garden AL, Hlynsson VF, Björgvinsdóttir S, Ólafsdóttir H, Skúlason E (2015). Phys. Chem. Chem. Phys..

[26] Abghoui Y, Skúlason E (2017). Catal. Today.

[27] Abghoui Y, Garden AL, Howalt JG, Vegge T, Skúlason E (2015). ACS Catal..

[28] Montoya JH, Tsai C, Vojvodic A, Nørskov JK (2015). Chemsuschem.

[29] Howalt JG, Vegge T (2014). Beilstein J. Nanotechnol..

[30] Höskuldsson AB, Abghoui Y, Gunnarsdóttir AB, Skúlason E (2017). ACS Sustain. Chem. Eng..

[31] Azofra LM, Li N, MacFarlane DR, Sun C (2016). Energy Environ. Sci..

[32] Liu Y, Su Y, Quan X, Fan X, Chen S, Yu H, Zhao H, Zhang Y, Zhao J (2018). ACS Catal..

[33] Zhang L, Ji X, Ren X, Ma Y, Shi X, Tian Z, Asiri AM, Chen L, Tang B, Sun X (2018). Adv. Mater..

[34] Zhang L, Ji X, Ren X, Luo Y, Shi X, Asiri AM, Zheng B, Sun X (2018). ACS Sustain. Chem. Eng..

[35] Yang X, Nash J, Anibal J, Dunwell M, Kattel S, Stavitski E, Attenkofer K, Chen JG, Yan Y, Xu B (2018). J. Am. Chem. Soc..

[36] Song Y, Johnson D, Peng R, Hensley DK, Bonnesen PV, Liang L, Huang J, Yang F, Zhang F, Qiao R, Baddorf AP (2018). Sci. Adv..

[37] Nazemi M, El-Sayed MA (2018). J. Phys. Chem. Lett..

[38] Yao, Y., Feng, Q., Zhu, S., Li, J., Yao, Y., Wang, Y.,… & Yuan, X. Z. (2018). Small Methods, 1800324

[39] Ran J, Zhang J, Yu J, Jaroniec M, Qiao SZ (2014). Chem. Soc. Rev..

[40] Xu L, Jiang Q, Xiao Z, Li X, Huo J, Wang S, Dai L (2016). Angew. Chem. Int. Ed..

[41] Ong WJ, Tan LL, Ng YH, Yong ST, Chai SP (2016). Chem. Rev..

[42] Fuertes A (2015). Mater. Horizons.

[43] Cao S, Low J, Yu J, Jaroniec M (2015). Adv. Mater..

[44] Zheng Y, Liu J, Liang J, Jaroniec M, Qiao SZ (2012). Energy Environ. Sci..

[45] Ran J, Gao G, Li FT, Ma TY, Du A, Qiao SZ (2017). Nat. Commun..

[46] Sun S, An Q, Wang W, Zhang L, Liu J, Goddard WA (2017). J. Mater. Chem. A.

[47] Li H, Shang J, Shi J, Zhao K, Zhang L (2016). Nanoscale.

[48] Bai Y, Ye L, Chen T, Wang L, Shi X, Zhang X, Chen D (2016). ACS Appl. Mater. Interfaces.

[49] Hirakawa H, Hashimoto M, Shiraishi Y, Hirai T (2017). J. Am. Chem. Soc..

[50] Zhao Y, Zhao Y, Waterhouse GI, Zheng L, Cao X, Teng F, Wu LZ, Tung CH, O’Hare D, Zhang T (2017). Adv. Mater..

[51] Dong G, Ho W, Wang C (2015). J. Mater. Chem. A.

[52] Zhang N, Jalil A, Wu D, Chen S, Liu Y, Gao C, Ye W, Qi Z, Ju H, Wang C, Wu X (2018). J. Am. Chem. Soc..

[53] Yang J, Guo Y, Jiang R, Qin F, Zhang H, Lu W, Wang J, Yu JC (2018). J. Am. Chem. Soc..

[54] Yang D, Chen T, Wang Z (2017). J. Mater. Chem. A.

[55] Bao D, Zhang Q, Meng FL, Zhong HX, Shi MM, Zhang Y, Yan JM, Jiang Q, Zhang XB (2017). Adv. Mater..

[56] Shi MM, Bao D, Wulan BR, Li YH, Zhang YF, Yan JM, Jiang Q (2017). Adv. Mater..

[57] Li SJ, Bao D, Shi MM, Wulan BR, Yan JM, Jiang Q (2017). Adv. Mater..

[58] Kim, K., Yoo, C.-Y., Kim, J.-N., Yoon, H.C. & Han, J.-I. **163**, F1523–F1526 (2016)

[59] Furuya N, Yoshiba H (1989). J. Electroanal. Chem. Interfacial Electrochem..

[60] Furuya N, Yoshiba H (1990). J. Electroanal. Chem. Interfacial Electrochem..

[61] Tsuneto A, Kudo A, Sakata T (1994). J. Electroanal. Chem..

[62] Kordali, V., Kyriacou, G. & Lambrou, C. Chem. Commun., 1673–1674 (2000)

[63] Köleli F, Röpke T (2006). Appl. Catal. B.

[64] Lan R, Irvine JTS, Tao S (2013). Sci. Rep..

[65] Lan R, Tao S (2013). RSC Adv..

[66] Kugler K, Luhn M, Schramm JA, Rahimi K, Wessling M (2015). Phys. Chem. Chem. Phys..

[67] Kim K, Lee N, Yoo CY, Kim JN, Yoon HC, Han JI (2016). J. Electrochem. Soc..

[68] Kong J, Lim A, Yoon C, Jang JH, Ham HC, Han J, Nam S, Kim D, Sung YE, Choi J, Park HS (2017). ACS Sustain. Chem. Eng..

[69] Chen S, Perathoner S, Ampelli C, Mebrahtu C, Su D, Centi G (2017). ACS Sustain. Chem. Eng..

[70] Chen GF, Cao X, Wu S, Zeng X, Ding LX, Zhu M, Wang H (2017). J. Am. Chem. Soc..

[71] Zhao X, Yin F, Liu N, Li G, Fan T, Chen B (2017). J. Mater. Sci..

[72] Chen S, Perathoner S, Ampelli C, Mebrahtu C, Su D, Centi G (2017). Angew. Chem. Int. Ed..

[73] Zhou F, Azofra LM, Ali M, Kar M, Simonov AN, McDonnell-Worth C, Sun C, Zhang X, MacFarlane DR (2017). Energy Environ. Sci..

[74] Liu HM, Han SH, Zhao Y, Zhu YY, Tian XL, Zeng JH, Jiang JX, Xia BY, Chen Y (2018). J. Mater. Chem. A.

[75] Ren X, Cui G, Chen L, Xie F, Wei Q, Tian Z, Sun X (2018). Chem. Commun..

[76] Zhang X, Kong R-M, Du H, Xia L, Qu F (2018). Chem. Commun..

[77] Sheets BL, Botte GG (2018). Chem. Commun..

[78] Lv C, Yan C, Chen G, Ding Y, Sun J, Zhou Y, Yu G (2018). Angew. Chem..

[79] Nazemi M, Panikkanvalappil SR, El-Sayed MA (2018). Nano Energy.

[80] Zhang C, Chen G, Lv C, Yao Y, Xu Y, Jin X, Meng Q (2018). ACS Sustain. Chem. Eng..

